# Personal

**Published:** 1899-12

**Authors:** 


### PERSONAL.

Dr. William G. Bissell, of Buffalo, Major and Surgeon of the 74th Regiment, N. Y. N. G., recently tendered his resignation of that office. He has been obliged to give great and increasing attention to the duties pertaining to it, which materially interfered with his professional work. He has recently been appointed lecturer on bacteriology in the Dental Department of the University of Buffalo, which makes still further demand upon his time. Since the foregoing was announced in the newspaper press, it is stated that Major Bissell has temporarily withdrawn his resignation at the request of Col. Fox, Lieut.-Col. Cottle and other officers of the regiment and will continue with the 74th as surgeon until the regiment is settled in its new armory.

---

Dr. Maud J. Frye, of Buffalo, one of the associate editors of the Journal, began instruction to the class in home-nursing and training attendants, at the Women's Union, November 6, 1899. The course embraces lectures on the following practical subjects: Sick-room, sick-bed, bath, diet of children, cookery, enemata, douches and catheterisation, sleep, taking the pulse, temperature and respiration, entertaining the patient, poultice making, etc., administration of medicines, bandaging, convalescence after confinement, emergencies, quarantine and disinfection, care of baby and massage.

---

Dr. Edward J. Meyer, who underwent an operation at the General Hospital a short time ago, has entirely recovered and resumed practice, at No. 1312 Main street. Dr. Meyer has given up general medical practice and is now confining himself to surgery.

---

Dr. C. G. Stivers, formerly of Niagara Falls, assistant editor of the *Southern California Practitioner*, has been appointed instructor in physical diagnosis in the Medical Department of the University of Southern California, at Los Angeles.

---

Dr. Charles St. John, formerly of the Fitch Hospital, has been appointed house surgeon of the Riverside Hospital.

